# Dietary amino acid profile and risk of hypertension: findings from the Ravansar cohort study

**DOI:** 10.1186/s40795-024-00878-2

**Published:** 2024-05-02

**Authors:** Farid Najafi, Parisa Mohseni, Mahdieh Niknam, Yahya Pasdar, Neda Izadi

**Affiliations:** 1 Research Center for Environmental Determinants of Health (RCEDH), Health Institute, Kermanshah University of Medical Sciences, Kermanshah, Iran; 2 Cellular and Molecular Research Center, Grash University of Medical Sciences, Gerash, Iran; 3 Research Center for Social Determinants of Health, Research Institute for Endocrine Sciences, Shahid Beheshti University of Medical Sciences, Tehran, Iran; 4 Research Center for Environmental Determinants of Health (RCEDH), Nutritional Science Department, Kermanshah University of Medical Sciences, Kermanshah, Iran

**Keywords:** Amino acid, Dietary intake, FFQ, Hypertension, Incidence, PERSIAN Cohort

## Abstract

**Introduction:**

Hypertension (HTN) is a significant global health concern associated with morbidity and mortality. Recent research has explored the potential relationship between dietary protein intake and the development of HTN. This study aims to investigate the association between dietary amino acids and the incidence of HTN.

**Methods:**

This nested case-control study utilized data from the Ravansar Non-Communicable Disease (RaNCD) Cohort Study. The study included 491 new HTN cases identified over a 6-year follow-up period. For each case, four controls were randomly selected through density sampling. A food frequency questionnaire (FFQ) consisting of 125 food items was used to calculate dietary amino acid intake. HTN was determined based on systolic blood pressure ≥ 140 mmHg and/or diastolic blood pressure ≥ 90 mmHg and/or current use of antihypertensive medication in subjects without pre-existing HTN at the start of the cohort study. Conditional logistic regression was used to estimate crude and adjusted odds ratios for HTN risk.

**Results:**

The median intake of all amino acids was lower in patients with HTN compared to the control group. After adjusting for various variables in different models, the risk of developing HTN tended to increase with higher dietary amino acid intake (excluding tryptophan and acidic amino acids). Specifically, individuals in the third tertile had a higher risk of developing new HTN than those individuals in the lowest tertile, although this difference was not statistically significant (*P* > 0.05).

**Conclusion:**

The findings suggest that there may be an association between increased dietary amino acid intake and the risk of developing HTN, although this association was not statistically significant in this study. Further investigations in diverse populations are needed to explore the relationship between amino acids and HTN, as well as to determine the potential positive and negative effects of specific amino acid patterns on hypertension.

## Introduction

Hypertension (HTN) is a significant contributor to global morbidity and mortality, as well as a leading cause of cardiovascular disease (CVD) [1]. Elevated blood pressure (BP) can lead to severe complications such as heart disease, heart failure, stroke, diabetes, visual impairment, and chronic kidney failure [2–4].

The prevalence of hypertension varies across regions and income groups worldwide. According to reports from the World Health Organization (WHO), Africa has the highest prevalence of HTN at 27%, while the Americas have the lowest at 18%. In addition, the number of adults with hypertension increased from 594 million in 1975 to 1.13 billion in 2015, primarily in low- and middle-income countries such as Iran [5, 6]. This rise can be attributed to the increase in risk factors for hypertension. Nutritional status and dietary intake play a crucial role in the incidence and severity of HTN, and there is growing interest in exploring the potential impact of specific nutrients on blood pressure regulation [7].

Amino acids have been recognized as significant components of dietary protein, which has been associated with various chronic diseases [8–10]. Recent studies have investigated the potential relationship between dietary protein intake and hypertension [11, 12]. While two cohort studies found no association between glutamic acid and HTN, a cross-sectional study reported an inverse association [13]. Similarly, conflicting results were observed for glycine in two cross-sectional studies [8, 14]. Contradictory findings were also noted for tyrosine, methionine, and alanine (8). The study by Altorf-van der Kuil (2013) emphasized the role of glutamic acid, arginine, lysine, tyrosine, and cysteine in determining hypertension risk [13]. In addition, Teymoori et al. (2017) found that high intakes of branched-chain, aromatic, proline, and alcoholic amino acids might elevate blood pressure [15]. Given the potential and controversial association between dietary amino acids and HTN, this study aims to investigate the link between dietary amino acids and the incidence of hypertension.

## Methods

### Study population

The present nested case-control study utilized data from the Ravansar Non-Communicable Disease (RaNCD) Cohort Study, which is a part of the PERSIAN (Prospective Epidemiological Research Studies in IrAN) Cohort. The RaNCD Cohort is a population-based prospective study consisting of individuals aged 35–65 years. Detailed information on this cohort can be found elsewhere [16].

**Diagram. 1:**
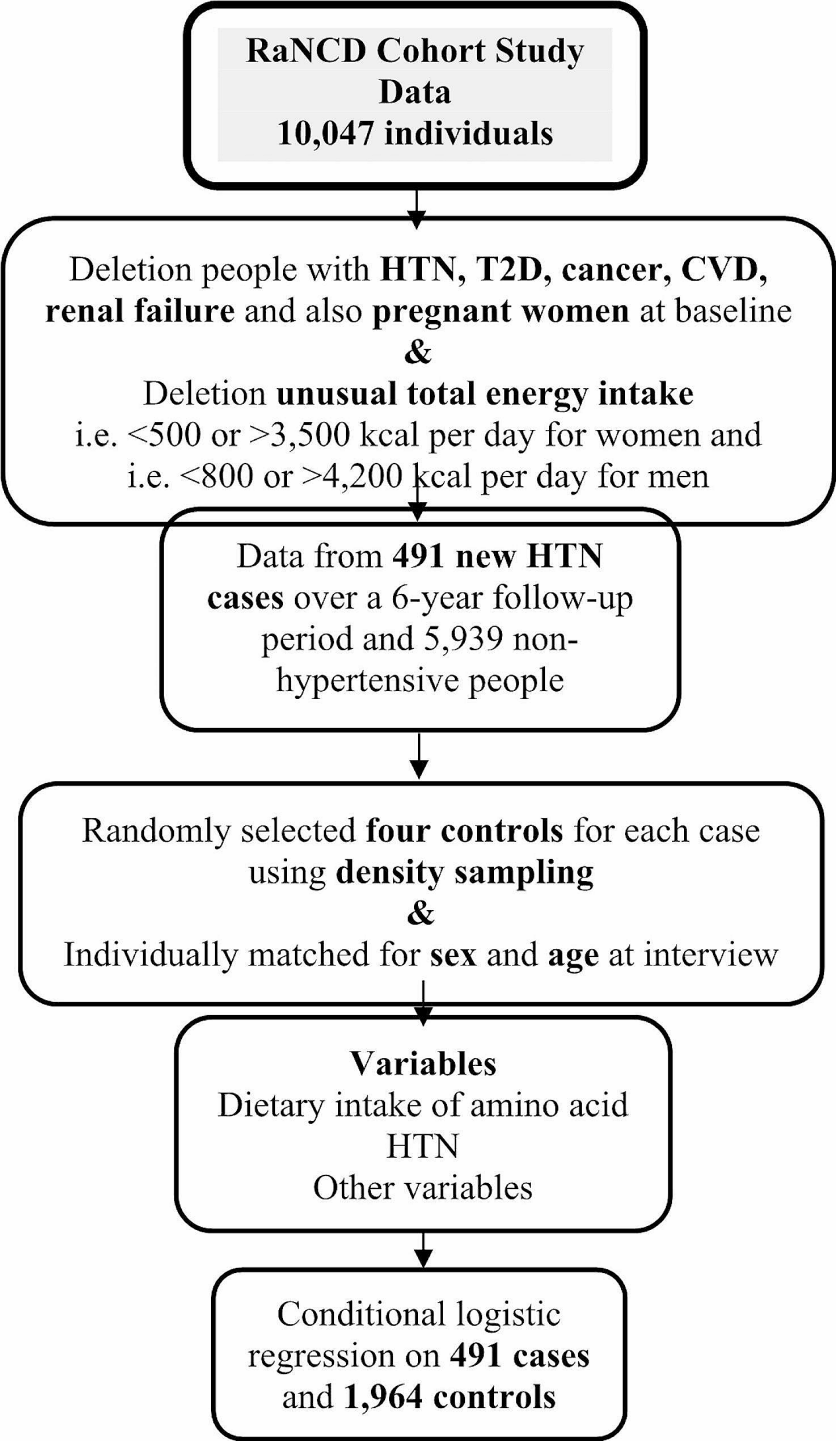
Flowchart of the study participants and data preparation

Data for this study were collected from participants who enrolled from the beginning of the study until September 2023. Participants with baseline diagnoses of hypertension (*n* = 1,442), cardiovascular disease (*n* = 495), type 2 diabetes (T2D) (*n* = 405), cancer (*n* = 67), pregnancy (*n* = 93), or renal failure (*n* = 37), as well as those with unusual total energy intake (< 500 or > 3,500 kcal per day for women and < 800 or > 4,200 kcal per day for men) [15, 17], were excluded from the dataset (*n* = 1,078). After exclusions, data from 491 incident cases of hypertension over a 6-year follow-up period were available for analysis. For each case, four controls were randomly selected using density sampling. Cases and controls were individually matched for sex and age (Diagram 1) All participants provided written informed consent, and the study was approved by the Kermanshah University of Medical Sciences Review Board.

### Data collection

#### Dietary intake of amino acids

Dietary intake of all amino acids, including tryptophan, threonine, isoleucine, leucine, lysine, methionine, cysteine, phenylalanine, tyrosine, valine, arginine, histidine, alanine, aspartic acid, glutamic acid, glycine, proline, and serine, was estimated using a Food Frequency Questionnaire (FFQ) consisting of 10 sections with 125 food items. Eghtesad et al. confirmed the validity and reproducibility of a food intake frequency questionnaire in the PERSIAN Cohort Study [18]. This questionnaire was used to determine the amount of common foods consumed by the participants. The amount of consumption of each amino acid was converted to grams per day (g/day) using Nutrition IV software to measure the daily intake of each amino acid. Amino acids were categorized into eight groups based on their chemical structure, including branched-chain amino acids (BCAAs) (leucine, isoleucine, valine), aromatic amino acids (AAA) (tryptophan, phenylalanine, tyrosine), alkaline (histidine, arginine, lysine), sulfuric (methionine, cysteine), acidic (glutamic acid, aspartic acid), alcoholic (serine, threonine), small amino acids (glycine, alanine), and cyclic side chains (proline). Additionally, two groups of essential (histidine, isoleucine, leucine, lysine, methionine, phenylalanine, threonine, tryptophan, valine) and non-essential (alanine, arginine, aspartic acid, cysteine, glutamic acid, glycine, proline, serine, tyrosine) amino acids were included in the model as major exposures. Tertiles were used to categorize the amino acid groups.

#### Hypertension

Sitting blood pressure, including systolic and diastolic measurements, was assessed using a standardized procedure after at least 10 min of rest. Hypertension was defined as systolic blood pressure (SBP) ≥ 140 mmHg and/or diastolic blood pressure (DBP) ≥ 90 mmHg and/or current use of antihypertensive medication among subjects who were free of illness at the start of the cohort study [19].

To control for confounding factors, several variables were considered, including education (years), place of residence (urban, rural), smoking status (current smoker, never smoker, passive smoker, former smoker), alcohol consumption (yes or no), physical activity (METs) (low (24–36.5 METs-h/day), moderate (36.6–44.4 METs-h/day), vigorous (≥ 44.5 METs-h/day)) [20, 21], socioeconomic status (SES) (poor, moderate, high), family history of HTN, dietary habits (healthy and unhealthy patterns, determined through principal component analysis of the FFQ), total energy intake (kcal), anthropometric characteristics (weight, height, body mass index (BMI), waist circumference (WC), waist-to-height ratio (WHtR)), metabolic syndrome (ATP definition) [22], and dyslipidemia (defined as total cholesterol (TC) ≥ 240 mg/dL and/or triglycerides (TG) ≥ 200 mg/dL and/or low-density lipoprotein (LDL) cholesterol ≥ 160 mg/dL and/or high-density lipoprotein (HDL) cholesterol < 40 mg/dL and/or use of medication for dyslipidemia) [23]. Detailed information on data collection and measurements has been described elsewhere [24–28].

### Statistical analysis

Descriptive statistics, including mean (standard deviation), median (interquartile range = IQR), and number (percentage), were used to summarize quantitative and qualitative variables, respectively. Conditional logistic regression was performed to estimate crude and adjusted odds ratios (ORs) for the risk of HTN. The models were adjusted for residence, SES, education, BMI, WHtR, smoking, alcohol consumption, physical activity, dietary habits, comorbidities, and daily energy intake. The data were analyzed using Stata software (version 15). A significance level of *P* < 0.05 was considered statistically significant for all statistical tests.

## Results

62.12% of individuals with HTN resided in urban areas. The frequency of poor SES was lower among individuals with hypertension compared to the control group (34.29% versus 38.95%). The frequency of current smokers was 10.06% among HTN patients and 12.16% in the control group. Alcohol consumption was similar between the case and control groups (2.26%). The proportion of individuals with low physical activity was higher among HTN patients than in the control group (31.98%). Mean anthropometric indices were also higher in patients with hypertension compared to those without hypertension. The frequency of family history of hypertension in patients was higher than in the control group (52.34% vs. 43.89%, respectively). Moreover, the frequency of comorbidities was higher among those with HTN **(**Table 1**)**.

The results indicated that the median values of all amino acids were lower in patients with HTN compared to the control group. Among the dietary amino acids, tryptophan had the lowest median intake (0.48 g/day in hypertension patients and 0.49 g/day in the control group), while glutamic acid had the highest median intake (8.23 g/day in hypertension patients and 8.25 g/day in the control group) **(**Table 2**)**.

In the univariable model, both individual amino acids and groups of eight amino acids, as well as essential and nonessential amino acids, showed a non-significant inverse association with the occurrence of hypertension. Furthermore, after adjusting for several variables in different models, the risk of developing HTN increased with higher dietary amino acid intake (except for tryptophan and acidic amino acids). Individuals in the third tertile had a higher risk of developing HTN compared to those in the lowest tertile, but these findings were not statistically significant (*P* > 0.05) **(**Table 3**)**.

**Table 1:** Frequency and distribution of different variables by group (case & control) in the Ravansar non-communicable disease cohort study

Variables		Case (*n* = 491)	Control (*n* = 1,964)
		*N* (%)	
**Education (year)†**		4.54 (4.73)	4.33 (4.60)
**Residency**			
	Urban	305 (62.12)	1064 (54.18)
	Rural	186 (37.88)	900 (45.82)
**Socio-economic status**			
	Poor	168 (34.29)	765 (38.95)
	Moderate	167 (34.08)	643 (32.74)
	High	155 (31.63)	556 (28.31)
**Smoke status**			
	Non**-**smoker	206 (42.30)	794 (40.93)
	Current smoker	49 (10.06)	236 (12.16)
	Former smoker	34 (6.98)	153 (7.89)
	Passive smoker	198 (40.66)	757 (39.02)
**Alcohol consumption**			
	Yes	16 (3.26)	64 (3.26)
	No	475 (96.74)	1900 (96.74)
**Physical activity (METs h/day)**			
	Low	157 (31.98)	537 (27.34)
	Moderate	264 (53.77)	987 (50.25)
	High	70 (14.26)	440 (22.40)
**Anthropometric indices†**			
	Body Mass Index, kg/m^2^	28.41 (4.45)	27.07 (4.66)
	Waist Circumference, cm	99.51 (9.45)	96.85 (10.29)
	Waist-to-height ratio	0.95 (0.05)	0.93 (0.06)
**Healthy pattern (dietary)**			
	Tertile 1	158 (32.18)	661 (33.66)
	Tertile 2	173 (35.23)	645 (32.84)
	Tertile 3	160 (32.59)	658 (33.50)
**Unhealthy pattern (dietary)**			
	Tertile 1	182 (37.07)	637 (32.43)
	Tertile 2	173 (35.23)	645 (32.84)
	Tertile 3	136 (27.70)	682 (34.73)
**Family history of hypertension (yes)**		257 (52.34)	862 (43.89)
**Comorbidities (yes)**			
	Dyslipidemia	210 (42.77)	717 (36.51)
	Metabolic syndrome (ATP)	123 (25.15)	308 (15.79)

^†^Mean (Standard deviation)

**Table 2:** Distribution of dietary amino acid profile by group in the Ravansar non-communicable disease cohort study

Amino Acids	Case (*n* = 491)	Control (*n* = 1,964)
	Median (IQR) (g/day)	
Tryptophan	0.48 (0.29)	0.49 (0.31)
Threonine	1.67 (1.06)	1.71 (1.12)
Isoleucine	1.95 (1.24)	2.00 (1.30)
Leucine	3.21 (2.05)	3.35 (2.13)
Lysine	2.84 (1.95)	2.88 (1.98)
Methionine	0.93 (0.63)	0.95 (0.63)
Cysteine	0.60 (0.35)	0.60 (0.37)
Phenylalanine	1.92 (1.19)	1.94 (1.20)
Tyrosine	1.43 (0.88)	1.46 (0.94)
Valine	2.29 (1.43)	2.37 (1.49)
Arginine	2.55 (1.64)	2.64 (1.73)
Histidine	1.12 (0.73)	1.15 (0.76)
Alanine	2.12 (1.42)	2.18 (1.43)
Aspartic acid	4.45 (2.73)	4.53 (2.88)
Glutamic acid	8.23 (4.67)	8.25 (4.90)
Glycine	1.71 (1.17)	1.76 (1.19)
Proline	2.41 (1.43)	2.39 (1.54)
Serine	1.98 (1.22)	2.04 (1.25)
Branched chain	7.48 (4.70)	7.74 (4.98)
Aromatic	3.84 (2.37)	3.93 (2.47)
Alkaline	6.54 (4.21)	6.71 (4.50)
Sulfuric	1.55 (0.97)	1.57 (1.00)
Acidic	12.61 (7.47)	12.81 (7.80)
Alcoholic	3.66 (2.25)	3.77 (2.39)
Small amino acid	3.82 (2.59)	3.95 (2.62)
Proline	2.41 (1.43)	2.39 (1.54)
Essential	16.46 (10.42)	16.95 (11.01)
Non-essential	25.52 (15.32)	25.83 (16.10)
Total	41.90 (26.27)	42.80 (27.01)

**Table 3:** Crude and adjusted association of HTN with dietary amino acid tertiles in the Ravansar non-communicable disease cohort study after 6 years of follow-up

Amino Acids	Model 1	Model 2	Model 3
	OR (95% CI)		
**Tryptophan**			
T2	1.10 (0.86–1.40)	1.12 (0.86–1.47)	1.10 (0.83–1.47)
T3	0.89 (0.68–1.16)	0.98 (0.70–1.37)	0.97 (0.65–1.43)
**Threonine**			
T2	1.03 (0.81–1.32)	1.07 (0.81–1.39)	1.05 (0.79–1.40)
T3	0.94 (0.72–1.23)	1.05 (0.75–1.46)	1.06 (0.73–1.56)
**Isoleucine**			
T2	1.02 (0.80–1.30)	1.04 (0.80–1.36)	1.04 (0.78–1.38)
T3	0.96 (0.74–1.25)	1.08 (0.78–1.50)	1.10 (0.76–1.60)
**Leucine**			
T2	1.06 (0.83–1.35)	1.09 (0.83–1.42)	1.09 (0.81–1.43)
T3	0.97 (0.74–1.26)	1.12 (0.80–1.55)	1.16 (0.79–1.70)
**Lysine**			
T2	0.98 (0.76–1.25)	0.99 (0.76–1.30)	0.99 (0.74–1.31)
T3	0.94 (0.72–1.22)	1.02 (0.74–1.41)	1.04 (0.71–1.50)
**Methionine**			
T2	1.08 (0.85–1.37)	1.12 (0.85–1.45)	1.12 (0.85–1.47)
T3	0.94 (0.72–1.22)	1.04 (0.75–1.45)	1.06 (0.74–1.53)
**Cysteine**			
T2	1.14 (0.89–1.45)	1.19 (0.91–1.56)	1.17 (0.88–1.55)
T3	0.98 (0.75–1.28)	1.15 (0.82–1.62)	1.17 (0.79–1.74)
**Phenylalanine**			
T2	1.08 (0.85–1.38)	1.10 (0.84–1.45)	1.09 (0.82–1.45)
T3	0.94 (0.72–1.22)	1.08 (0.77–1.51)	1.10 (0.75–1.62)
**Tyrosine**			
T2	1.00 (0.78–1.28)	1.02 (0.78–1.33)	1.01 (0.76–1.34)
T3	0.93 (0.71–1.21)	1.04 (0.75–1.44)	1.06 (0.73–1.55)
**Valine**			
T2	0.97 (0.76–1.24)	0.98 (0.75–1.29)	0.97 (0.73–1.30)
T3	0.94 (0.72–1.22)	1.03 (0.74–1.44)	1.05 (0.71–1.53)
**Arginine**			
T2	1.06 (0.83–1.35)	1.10 (0.84–1.45)	1.09 (0.81–1.45)
T3	0.94 (0.73–1.23)	1.06 (0.76–1.50)	1.08 (0.73–1.60)
**Histidine**			
T2	0.99 (0.77–1.26)	1.01 (0.77–1.32)	0.99 (0.74–1.31)
T3	0.94 (0.73–1.23)	1.02 (0.73–1.41)	1.02 (0.70–1.49)
**Alanine**			
T2	0.99 (0.78–1.27)	1.03 (0.79–1.35)	1.01 (0.76–1.35)
T3	0.96 (0.74–1.25)	1.05 (0.75–1.47)	1.06 (0.72–1.56)
**Aspartic acid**			
T2	0.99 (0.78–1.27)	1.05 (0.80–1.38)	1.05 (0.78–1.41)
T3	0.96 (0.74–1.25)	1.07 (0.76–1.51)	1.12 (0.74–1.69)
**Glutamic acid**			
T2	0.96 (0.75–1.22)	0.97 (0.74–1.27)	0.95 (0.71–1.27)
T3	0.90 (0.70–1.17)	0.98 (0.70–1.38)	1.01 (0.68–1.50)
**Glycine**			
T2	1.03 (0.81–1.31)	1.10 (0.84–1.44)	1.07 (0.81–1.42)
T3	0.93 (0.72–1.21)	1.03 (0.74–1.43)	1.03 (0.71–1.50)
**Proline**			
T2	0.94 (0.74–1.20)	0.97 (0.74–1.28)	0.97 (0.72–1.28)
T3	0.91 (0.70–1.18)	0.99 (0.71–1.38)	1.04 (0.71–1.53)
**Serine**			
T2	0.92 (0.72–1.18)	0.91 (0.69–1.20)	0.89 (0.66–1.18)
T3	0.92 (0.71–1.20)	1.04 (0.74–1.46)	1.08 (0.71–1.58)
**Branched chain**			
T2	1.07 (0.84–1.36)	1.11 (0.85–1.45)	1.11 (0.83–1.47)
T3	0.98 (0.76–1.28)	1.14 (0.82–1.59)	1.20 (0.81–1.73)
**Aromatic**			
T2	1.06 (0.83–1.36)	1.09 (0.83–1.43)	1.08 (0.81–1.43)
T3	0.96 (0.74–1.25)	1.11 (0.79–1.54)	1.14 (0.78–1.67)
**Alkaline**			
T2	1.02 (0.80–1.31)	1.05 (0.80–1.38)	1.04 (0.78–1.38)
T3	0.95 (0.73–1.24)	1.05 (0.75–1.47)	1.07 (0.73–1.58)
**Sulfuric**			
T2	1.13 (0.88–1.44)	1.16 (0.89–1.52)	1.15 (0.87–1.52)
T3	0.96 (0.74–1.26)	1.08 (0.77–1.51)	1.10 (0.75–1.61)
**Acidic**			
T2	1.06 (0.83–1.35)	1.06 (0.81–1.39)	1.03 (0.77–1.37)
T3	0.89 (0.68–1.15)	0.95 (0.67–1.33)	0.94 (0.63–1.42)
**Alcoholic**			
T2	0.99 (0.77–1.26)	0.99 (0.75–1.30)	0.98 (0.74–1.30)
T3	0.94 (0.73–1.23)	1.05 (0.75–1.47)	1.08 (0.73–1.60)
**Small amino acid**			
T2	1.02 (0.80–1.30)	1.08 (0.83–1.42)	1.06 (0.80–1.41)
T3	0.96 (0.74–1.25)	1.07 (0.77–1.49)	1.08 (0.74–1.59)
**Proline**			
T2	0.94 (0.74–1.20)	0.97 (0.74–1.28)	0.96 (0.72–1.28)
T3	0.91 (0.70–1.18)	0.99 (0.71–1.38)	1.04 (0.71–1.53)
**Essential**			
T2	1.00 (0.78–1.28)	1.04 (0.79–1.36)	1.03 (0.78–1.37)
T3	0.95 (0.73–1.24)	1.07 (0.77–1.49)	1.10 (0.75–1.60)
**Non-essential**			
T2	1.02 (0.80–1.30)	1.03 (0.79–1.36)	1.01 (0.76–1.35)
T3	0.93 (0.72–1.22)	1.02 (0.72–1.43)	1.02 (0.69–1.52)
**Total**			
T2	1.01 (0.79–1.28)	1.03 (0.79–1.35)	1.02 (0.77–1.36)
T3	0.95 (0.73–1.24)	1.06 (0.76–1.49)	1.09 (0.74–1.61)

**Model 1**: unadjusted model; **Model 2**: adjusted for residency (urban, rural), education, SES (poor, moderate and high), BMI, WHtR, smoking (non-smoker/passive smoker, current/former smoker), and daily energy intake; **Model 3**: adjusted for model 2 and physical activity (METs) (low, moderate and high), family history of hypertension, dietary patterns (healthy or unhealthy), and comorbidities (yes or no); **OR =** Odds Ratio; **CI =** Confidence Interval

## Discussion

Based on the available studies and the results of this particular study, the relationship between dietary amino acids and hypertension is complex and contradictory. Different studies have reported varying associations between specific amino acids and hypertension risk. Some studies have shown positive associations, while others have shown negative or no significant associations [29–31].

In this study, the highest median dietary amino acid was glutamic acid, which was consistent with findings from other studies. However, the results did not reach statistical significance in terms of the association between amino acid intake and hypertension risk.

In the International study of Macro/Micronutrients and Blood Pressure (INTERMAP) and the Rotterdam study [13, 32], glutamic acid was the most important amino acid in the diet. Whereas in the study by Javidan et al. [33], lysine was the most important amino acid in the diet and glutamic acid was consumed little.

The relationship between branched-chain amino acids (BCAAs) and hypertension risk has also been examined, with conflicting results. Some studies have reported positive associations, while others have found no significant associations. The same applies to other amino acids such as phenylalanine, methionine, alanine, tyrosine, and glycine.

The results of a study in the Chinese population showed a nonlinear relationship between BCAA intake and hypertension risk [34]. A cross-sectional study in Caucasian men showed that plasma BCAA levels were positively associated with HTN [35]. However, another Japanese study found that the association between plasma BCAAs and hypertension was abolished [36]. Hu et al., also reported that serum BCAAs were not associated with SBP or DBP [29].

In a study in a Dutch population, no association was found between usual intake of glutamic acid, arginine, lysine, and cysteine (expressed as a percentage of protein) and hypertension, and a borderline inverse association was observed between tyrosine intake and SBP, but not DBP. None of the amino acids was associated with a 6-year risk of hypertension [13].

In contrast to our study, the study by Stammler et al., reported a favorable association between glutamic acid intake and BP and showed that high glutamic acid intake was associated with lower systolic and diastolic blood pressure levels [32].

In a study group of Iranian adults, dietary intake of tyrosine and tryptophan showed no effect on BP after 3 years of follow-up. Only phenylalanine intake in the highest quartile was associated with a significantly increased risk of hypertension compared with the lowest quartile. However, when considered as a whole, total dietary AAA intake was positively and significantly associated with an increased risk of developing HTN [30].

In a large population-based cohort study, plasma essential amino acids leucine, valine, and isoleucine were associated with the occurrence of HTN in adults of predominantly European ancestry [37]. In a longitudinal study conducted in an Iranian group, dietary BCAAs clustered with AAA and proline showed a positive association with the incidence of HTN [15]. In a study by Mirmiran et al., higher BCAA intake was associated with a higher risk of HTN in a fully adjusted model [38]. Similar results of a positive relationship between BCAAs and AAA were found in two Asian studies [39].

In the study by Tuttle et al., higher dietary intake of methionine and alanine was associated with an increased risk of higher BP levels, whereas higher threonine and histidine intake showed an inverse association with BP [40]. In addition, Teymoori et al., found a direct association between dietary phenylalanine intake and HTN in a fully adjusted model, with a reciprocal adjustment for other amino acids [30]. Another study showed that plasma phenylalanine along with BCAA was positively related to systolic and diastolic blood pressure [35].

Previous studies have reported that methionine and alanine increase the risk of HTN [40]. An increase in dietary methionine consumption has been associated with an increase in SBP and DBP [30, 40]. Methionine is an essential amino acid. Among its metabolic byproducts, homocysteine, when elevated, is known for its ability to impair endothelial function and induce the production of asymmetric dimethylarginine (ADMA), which in turn can inhibit nitric oxide synthesis [41]. Thus, the effects of methionine on BP are indirect and occur through an increase in homocysteine levels, as shown by studies with methionine supplements in animals and humans [42, 43].

Several amino acids interfere with vascular physiology. Arginine is known to have vasogenic properties [44]. Studies that focused on dietary arginine and considered only a typical diet, with the exception of supraphysiological intake via dietary supplements, have not shown an association between this amino acid and BP [45, 46]. However, dietary supplementation with arginine has been shown to be beneficial in lowering systolic and diastolic blood pressure in hypertensive patients [47]. Conflicting results have been reported by epidemiological studies [13, 32, 33].

In one study, consumption of alcoholic amino acids (serine and threonine), classified together with BCAA and AAA as dietary patterns, with interactions and synergistic effects between these three groups of amino acids, increased the risk of HTN by 83% [15]. In addition, one study showed that serine consumption in the Iranian population increased the risk of hypertension by 70% [48].

Study by Fernstrom et al., suggested that tyrosine may act as a precursor to norepinephrine in the brain, reducing sympathetic tone and thus lowering BP [49]. However, in a randomized double-blind trial of 13 adults with mild hypertension, two weeks of supplementation with 7.5 g/day of tyrosine had no effect on HTN [50].

In the INTERMAP study, dietary glycine intake (expressed as a percentage of total protein and based on a 24-hour recall) was positively associated with SBP and DBP [32]. The opposite results may be supported by the important role of glycine in reducing oxidative stress and supporting nitric oxide function. In addition, glycine is involved in the synthesis of structural proteins such as elastin. Alteration in elastin formation have been associated with impaired vascular elastic properties, an important aspect in the pathogenesis of HTN [51]. In a study of a Mediterranean male population, plasma glycine levels showed a negative relationship with SBP [52]. In contrast to our findings, one study showed a protective relationship between dietary cysteine and HTN, with cysteine being able to mitigate BP by reducing oxidative stress, increasing nitric oxide bioavailability, and improving insulin sensitivity [15].

The mechanisms underlying the association between amino acids and HTN are not yet fully understood. They may involve various pathways, including the modulation of ion channels, protein synthesis, glucose metabolism, oxidative stress, and nitric oxide function [34, 53].

It’s important to note that the contradictory findings across studies may be attributed to methodological differences, population variations, dietary sources, and the complexity of amino acid interactions. Therefore, it is difficult to draw definitive conclusions regarding the effects of amino acid intake on HTN in humans.

Several limitations of our study should be mentioned. First, because our study was conducted in an Iranian population, such results may not be well generalized to other populations in different contexts. Second, our results only show that in the case of a relatively high level of dietary amino acid intake, the adverse effects of increasing the risk of hypertension can be observed. The main strengths of our study was its cohort design, which infers a causal relationship. In addition, this is a population-based study with adequate sample size and a wide age range (35–65 years) that is representative of the general population.

## Conclusions

The findings suggest that there may be an association between increased dietary amino acid intake and the risk of developing HTN, although this association was not statistically significant in this study. Further research is needed, including studies in diverse populations, to better understand the association between amino acids and hypertension and to determine the potential positive and negative effects of specific amino acid patterns on chronic diseases like hypertension.

## Data Availability

The datasets used and/or analyzed during the current study are available from the corresponding author on reasonable request.

## Abbreviations

AAA: Aromatic Amino Acids
BCAA: Branched-chain amino acids
BMI: Body mass index
BP: Blood pressure
CVD: Cardiovascular disease
DBP: Diastolic blood pressure
FFQ: Food frequency questionnaire
HDL: High-density lipoprotein
HTN: Hypertension
IQR: Interquartile range
LDL: Low-density lipoprotein
METs: Metabolic equivalents
OR: Odds ratio
RaNCD: Ravansar Non-Communicable Disease
SES: Socioeconomic status
SBP: Systolic blood pressure
T2D: Type 2 diabetes
TG: Triglyceride
WC: Waist circumference
WHtR: Waist-to-height ratio
